# Supplementary material for: A Score to Predict the Malignancy of a Breast Lesion Based on Different Contrast Enhancement Patterns in Contrast-Enhanced Spectral Mammography
Source: Cancers (Basel). 2022 Sep 5;14(17):4337. doi: 10.3390/cancers14174337 (PMC9455061; doi:10.3390/cancers14174337)

Table 1s. Summary of the histological results

Histological result	Overall (N=377)
Acinic cell carcinoma	2
Adenosis	11
Atypical ductal hyperplasia (DIN1b)	6
Atypical lobular hyperplasia (LIN1)	5
Breast fibroadenoma	10
Cribriform carcinoma	2
Ductal hyperplasia without atypia	6
Fibocistic disease	1
Fibroadenoma	2
Fibrocystic breast disease	55
Fibrosis	5
Flat epithelial atypia	3
Flogosis	7
High grade ductal carcinoma in situ (DIN3)	9
Intermediate grade ductal carcinoma in situ (DIN2)	18
Intermediate grade lobular intraepithelia neoplasia (LIN2)	1
Intraductal papilloma	6
Invasive cribriform carcinoma	2
Invasive ductal carcinoma	163
Invasive lobular carcinoma	33
Invasive mixed carcinoma	1
Lobular intraepithelial neoplasia (LIN2)	4
Low grade ductal carcinoma in situ (DIN1c)	7

Malignant phylloid	1
Micropapillary carcinoma	1
Mixed ductal and lobular carcinoma	9
Mucinous carcinoma	1
Mucocele like lesion	1
Papillomatosis	1
Pseudoangiomatous stromal hyperplasia (PASH)	1
Radial scar	3

Table 2s. Distribution of different enhancements and biopsy or surgery histological result (gold standard) (N=377)

		**Histological result**			
**Enhancements**		Benign lesion	Malignant (In situ) lesion	Malignant (Invasive) lesion	**Total**
**Intensity**	Benign lesion	116	15	38	169
	Malignant lesion	12	17	179	208
**Margin morphology**	Benign lesion	115	9	20	144
	Malignant lesion	13	23	197	233
**Pattern**	Benign lesion	121	11	31	163
	Malignant lesion	7	21	186	214
**Ground glass**	Benign lesion	117	13	33	163
	Malignant lesion	11	19	184	214
**Enhancement score ≥ 2**	No, Benign lesion	115	8	11	134
	Yes, Malignant lesion	13	24	206	243
	**Total**	128	32	217	377

Table 3s. Phi Coefficient among CESM enhancements (Benign vs. Malignant)

Enhancements	Intensity	Margin morphology	Pattern	Ground glass
**Intensity**		0.62	0.65	0.94
**Margin morphology**	0.62		0.76	0.67
**Pattern**	0.65	0.76		0.68
**Ground glass**	0.94	0.67	0.68	

Figure S1. ROC curve of the model with score enhancements as independent variable and histological result (Benign vs. Any malignant) as dependent variable

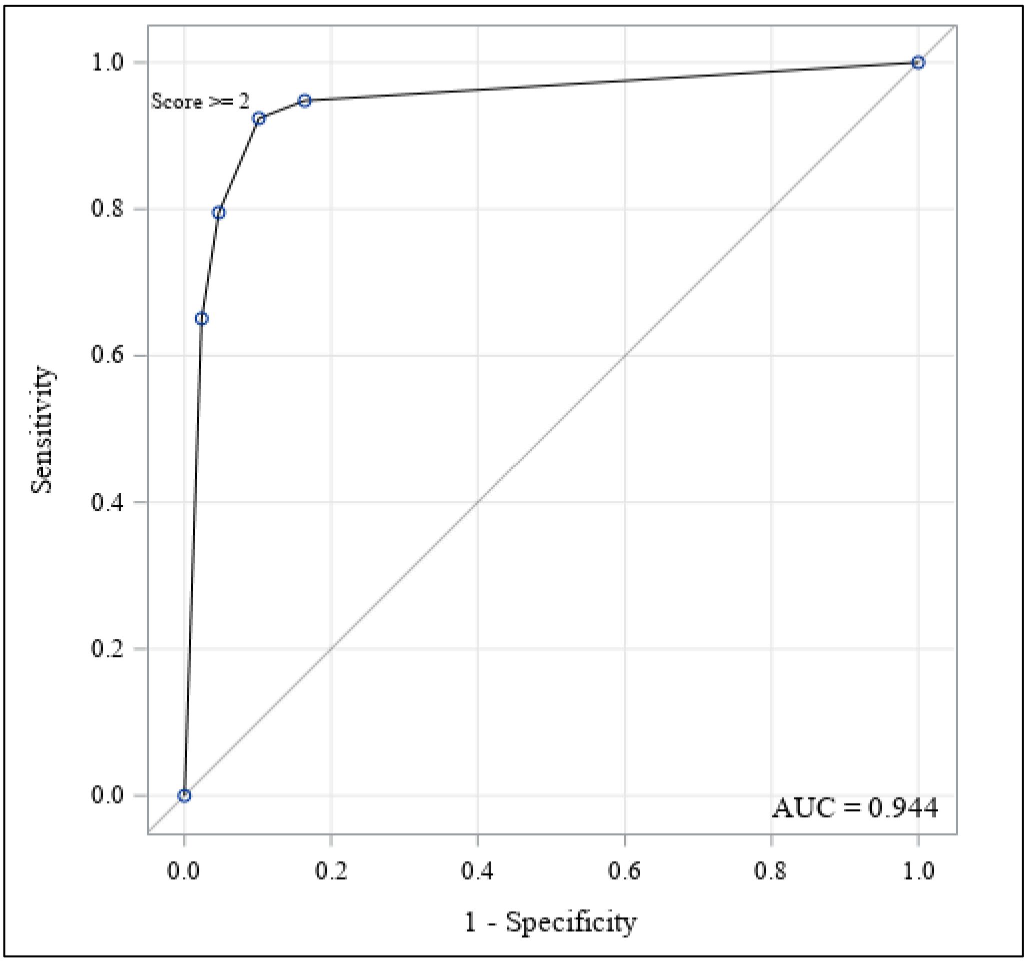

Supplement: Supplementary file 1 [file cancers-14-04337-s001.zip › cancers-1830906-supplementary.pdf]
